# Symptoms of prolonged grief, posttraumatic stress, and depression in recently bereaved people: symptom profiles, predictive value, and cognitive behavioural correlates

**DOI:** 10.1007/s00127-019-01776-w

**Published:** 2019-09-18

**Authors:** Paul A. Boelen, Lonneke I.M. Lenferink

**Affiliations:** 1 Department of Clinical Psychology, Faculty of Social Sciences, Utrecht University, PO Box 80140, 3508 TC Utrecht, The Netherlands; 2 Arq National Psychotrauma Centre, Nienoord 5, 1112 XE Diemen, The Netherlands; 3 Arq Centrum’45, Nienoord 5, 1112 XE Diemen, The Netherlands; 4 Department of Clinical Psychology and Experimental Psychopathology, Faculty of Behavioral and Social Sciences, University of Groningen, Grote Kruisstraat 2/1, 9712 TS Groningen, The Netherlands

**Keywords:** Bereavement, Grief, Posttraumatic stress, Prevention, LCA, PTSD, Depression

## Abstract

**Purpose:**

Prior latent class analyses (LCA) have focused on people who were bereaved more than 6 months earlier. Research has yet to examine patterns and correlates of emotional responses in the first few months of bereavement. We examined whether subgroups could be identified among very recently (≤ 6 months) bereaved adults, based on their endorsement of symptoms of prolonged grief disorder (PGD), posttraumatic stress disorder (PTSD), and depression. Associations of class membership with overall disturbed grief, PTSD, and depression—assessed concurrently and at 6 months follow-up—were examined. Furthermore, we examined differences between classes regarding socio-demographics, loss-related, and cognitive behavioural variables.

**Methods:**

PGD, PTSD, and depression self-report data from 322 Dutch individuals bereaved ≤ 6 months earlier were subjected to LCA; *N* = 159 completed the follow-up assessment. Correlates of class membership were examined.

**Results:**

Three classes were identified: a low symptom class (*N* = 114; 35.4%), a predominantly PGD class (*N* = 96; 29.8%), and a high symptom class (*N* = 112; 34.8%). PGD, PTSD, and depression scores (assessed concurrently and at 6 months follow-up) differed significantly between classes, such that low symptom class < predominantly PGD class < high symptom class. Being a woman, younger, more recently bereaved, experiencing deaths of a partner/child and unnatural losses, plus maladaptive cognitions and avoidance behaviours were associated with membership of the pervasive symptom classes.

**Conclusion:**

In the first 6 months of bereavement, meaningful subgroups of bereaved people can be distinguished, which highlights the relevance of early detection of people with elevated bereavement-related distress and offering them preventive interventions that foster adaptation to loss.

## Introduction

There is significant variation in how people respond to the death of a loved one. Many people experience little disruption in functioning; others experience initial distress declining in the first several months following loss. A minority of approximately 10% of bereaved individuals is plagued by persistent and disabling distress [1, 2]. This distress may manifest in symptoms of prolonged grief disorder (PGD; 3) or persistent complex bereavement disorder (PCBD) as included in DSM-5 [4]—including yearning, difficulties in accepting the loss, and moving on without the lost person—as well as depression, posttraumatic stress disorder (PTSD), other anxiety disorders, and suicidal ideation that are frequently comorbid with PGD/PCBD [5, 6].

There is gradually growing knowledge about risk factors for poor bereavement outcome. Research has shown that disturbed grief is associated with loss of closer loved ones, losses due to unnatural/violent causes, limited social support, and personality features of insecure attachment and elevated neuroticism [5, 7]. Yet, there is a need to enhance knowledge about risk factors. For instance, inconsistencies in research findings have been observed [5, 7]. These may be due to differences in study methods and samples and stress the importance of additional risk factor research. One issue that has particular theoretical and clinical relevance concerns the nature and correlates of responses to loss in the first months of bereavement. This is important because these initial responses have proven to be important predictors of later responses [6, 8].

In one recent study [8], we used latent class analysis (LCA) to examine if subgroups could be identified and characterized by similar symptom patterns of PCBD, among 476 people bereaved within the previous half year. LCA is a person-centred method that identifies classes of persons based on overlapping responses to a set of indicators (e.g. [9]). Our prior study showed that participants could be categorized into one group with no PCBD symptoms, a second group with elevated separation distress symptoms, and a third group with high severity of most PCBD symptoms. In addition, class membership was found to have prognostic value as evidenced by associations with PCBD severity and functional impairment assessed 3 years later. Drawing from cognitive behavioural conceptualizations of disturbed grief [10], we also examined to what extent classes differed in terms of seven cognitive behavioural variables. A first variable was a sense of unrealness about the irreversibility of the loss; four further variables were negative cognitions about the self, life, and the future, and catastrophic misinterpretations of grief reactions; two final variables were anxious avoidance of stimuli reminding of the loss and depressive avoidance of activities that could foster adjustment. Classes differed straightforwardly in terms of these maladaptive cognitive behavioural variables, such that these variables were lowest in the no PCBD symptom class, higher in the separation distress class, and highest in the high PCBD severity class.

The current study further examined patterns and correlates of early responses to loss. We expanded our prior study [8] by focusing on symptoms of PGD (as put forth by Prigerson et al. [3]) rather than PCBD, by examining classes based not only on symptoms of grief but also symptoms of PTSD and depression, and by evaluating the prognostic value of classes in predicting functioning 6 months rather than 3 years later. Criteria for PCBD as per DSM-5 and PGD as per Prigerson et al. [3] have attracted increasing attention from researchers; although these conditions are largely similar in terms of core symptoms, prevalence rates, and prognostic validity [11, 12], little, if anything, is, to the best of our knowledge, known about patterns and correlates of symptoms of PCBD and PGD among the very recently bereaved. Thus, the current study may expand our knowledge about these conditions. The reason to focus on a 6 months follow-up was that this allowed us to explore the association of early responses with responses surrounding the first anniversary of the loss, which is generally a period associated with elevated distress [13].

Thus, a first aim of the current study was to examine whether subgroups of people with different patterns of symptoms of PGD, PTSD, and depression could be identified among people who lost loved ones with the previous 6 months. Building on earlier work—prior to the LCA research—showing that PGD, PTSD, and depression are among the most commonly observed disorders following bereavement [14], several prior studies have identified subgroups of bereaved people based on symptoms of PGD/PCBD, PTSD, and/or depression. These studies mostly identified at least three classes, including a class with low symptom levels, a class with elevated PGD/PCBD (but not other) symptoms, and a class with elevated combinations of symptoms [15–21]. Based on this prior work, we expected different classes, including classes characterized by low symptoms, elevated PGD symptoms only, and elevated symptoms across all three symptom clusters.

The second aim was to examine whether subgroup membership predicted symptom levels of PGD, PTSD, and depression approximately 6 months after baseline. We anticipated that members of more disturbed classes would report more severe distress later in time. As a third aim, we explored correlates of subgroup membership. In so doing, we examined socio-demographic variables (age, gender, and educational level) and characteristics of the loss (time since loss, kinship to the deceased, and cause of loss) expecting membership of more disturbed classes to be predicted by lower education, the loss having a violent cause, and being a partner or parent of the deceased (cf. [5, 7]). Furthermore, we again considered the seven cognitive behavioural variables (i.e. unrealness, four domains of negative cognitions, and depressive and anxious avoidance). Based on prior research [8, 22] we predicted that, if LCA would reveal classes characterized by different levels of distress, people in the more disturbed classes would more strongly endorse these cognitive behavioural variables. Enhancing knowledge about the associations between different symptom patterns and these modifiable cognitive behavioural variables was considered to have clinical relevance, e.g. because it could inform the development of interventions targeting early signs of disturbed grief.

## Method

### Participants and procedure

This study drew from the Utrecht Longitudinal Study on Adjustment To Loss (ULSATL), an ongoing research programme on course and correlates of emotional consequences of bereavement [23, 24]. For this programme, participants were recruited via announcements on Internet sites. After completing an online application form, participants were sent a personal login code and referred to a secure online questionnaire. In the period of data collection for this study, 1797 people completed an application form and 906 (50.4%) completed the questionnaires. All participants provided informed consent. A local ethics committee approved this study. All people aged 18 years or older, who were able to read the information about the study, to provide informed consent, and to complete the questionnaires, were allowed to participate, with no further inclusion or exclusion criteria.

We selected data from 322 individuals bereaved ≤ 6 months before inclusion. The sample comprised 237 (73.6%) women. Participants had a mean age of 55.46 (SD = 14.03) years; 146 participants (45.3%) had had primary/secondary education and 176 (54.7%) had been to college or university. The mean time since loss was 3.40 (SD = 1.59) months; 150 (46.6%) participants had lost a spouse/partner, 21 (6.5%) a child, and 151 (46.9%) lost another relative. Losses were due to natural causes (e.g. illness, heart attack) in 294 cases (91.3%) and unnatural cause (suicide, accident, or homicide) in 28 cases (8.7%).

Of all 322 participants completing measures at time 1 (T1), 159 completed measures at a follow-up time (T2). Compared to the 163 not participating at T2, these 159 participants were older, included more people confronted with losses other than death of a partner of child, and had experienced their losses longer ago (all *p*s < 0.05); they did not differ on any of the other variables included in our analyses.

### Measures

#### Prolonged Grief Disorder Scale (PGD scale)

The PGD scale is an 11-item measure, based on the 19-item Inventory of Complicated Grief [25], tapping criteria for PGD as proposed by Prigerson et al. [3]. Accordingly, items represent one separation distress symptom, nine cognitive and emotional symptoms, and one functional impairment symptom. Participants rated how often symptoms occurred in the preceding month on 5-point scales (1 = never, 5 = always). Psychometric properties of the PGD scale are adequate [23]. In the current sample, Cronbach’s α was 0.92 at T1 and 0.93 at T2.

#### PTSD Symptom Scale—self-report version (PSS-SR)

The PSS-SR is a 17-item measure assessing PTSD as per DSM-IV [26]. Participants rated the frequency of symptoms during the preceding month on 4-point scales (0 = not at all, 3 = five/more times per week/almost always) keeping in mind their loss as the anchor event (e.g. “How often did you have unpleasant dreams or nightmares about the death of your loved one?”). English [27] and Dutch versions [28] have good psychometric properties. In the current sample, the α was 0.90 at T1 and 0.88 at T2.

#### Hospital Anxiety and Depression Scale—depression scale (HADS-D)

Depression symptoms were assessed with the depression scale of the HADS that asks respondents to rate the presence of seven depression symptoms during the preceding week on 4-point scales with anchors 0 and 3. English [29] and Dutch versions [30] have shown adequate psychometric properties. In the current sample, αs were 0.93 and 0.92 at T1 and T2, respectively.

#### Experienced Unrealness Scale

This is a five-item scale tapping a subjective sense of ambivalence about the irreversibility of the loss. Items (e.g. “It feels unreal that [–] is gone forever”) are rated on 8-point scales (1 = not at all true for me, 8 = completely true for me). Prior studies have supported the psychometric properties of the scale (e.g. [23]). In the present sample, the α was 0.93.

#### Grief Cognitions Questionnaire (GCQ) subscales Self, Life, Future, and Catastrophic Misinterpretations

The GCQ is a 38-item measure of negative bereavement-related cognitions [31]. We used four subscales tapping negative cognitions about the Self (six items, e.g. “Since [–] is dead, I am of no importance to anybody anymore”), Life (four items, e.g. “My life has no purpose anymore, since [–] died”), the Future (five items, e.g. “In the future I will never become really happy anymore”), and Catastrophic Misinterpretations of grief reactions (four items, e.g. “If I would fully realize what the death of [–] means, I would go crazy”), respectively. Items are rated on 6-point scales (1 = disagree strongly, 6 = agree strongly). Research has supported the measure’s psychometrics (e.g. [31]). In the present sample, the αs were: 0.92 (Self), 0.88 (Life), 0.86 (Future), and 0.88 (Catastrophic Misinterpretations).

#### Depressive and anxious avoidance in Prolonged Grief Questionnaire (DAAPGQ)

The DAAPGQ includes four items tapping Anxious Avoidance (e.g. “I avoid situations and places that confront me with the fact that [–] is dead and will never return”) and five items tapping Depressive Avoidance (e.g. “I avoid doing activities that used to bring me pleasure, because I feel unable to carry out these activities”). Items are rated on 8-point scales (1 = not at all true for me, 8 = completely true for me). Prior research has supported psychometric properties of the measure [32]. In the present sample, the αs were 0.77 (Anxious Avoidance) and 0.92 (Depressive Avoidance).

### Statistical analyses

We performed LCA using LatentGOLD (version 5.0.0, [33]) to identify the smallest number of unobserved classes of participants that can account for associations between dichotomously scored symptoms of PGD, PTSD, and depression. Due to limitations of the sample size, we selected six PTSD items from the PSS-SR corresponding to the six-item PTSD checklist—a validated brief screening checklist [34]. To obtain dichotomous indicators for each symptom, PGD symptoms were considered “absent” when rated with one, two, or three responses and “present” when rated with four or five responses. PTSD and depression symptoms were considered “absent” when rated 0 or 1 and “present” when rated 2 or 3.

A maximum of eight responses (2.5%) were missing for each indicator. Full maximum-likelihood estimation was used to deal with missing data. We first fitted the one-class model, followed by models with increasing numbers of classes to determine the number of latent classes that best fit the data. Statistical and non-statistical criteria were used to determine the optimal number of classes. With respect to statistical criteria we evaluated the (1) Akaike’s information criterion (AIC), (2) Bayesian information criterion (BIC), and (3) sample size-adjusted Bayesian information criterion (SS-BIC), with lower values indicating better model fit, plus (4) the entropy *R*^2^, with values closer to 1 indicating better class separation, and (5) the bootstrap likelihood ratio test (BLRt), a significant *p* value of which indicates that the model under consideration fits significantly better compared to the model with one less class [35]. With respect to non-statistical criteria, interpretability and size of latent classes were considered; solutions with theoretical meaningful classes were preferred, and solutions with relatively small class sizes were avoided due to potential computational difficulties in examining correlates of classes.

Correlates of class membership were examined using the three-step approach in LatentGOLD. Within this approach, the classification error, resulting from assigning people to classes with the highest probability estimate, is taken into account. To examine to what extent class membership predicted overall distress (i.e. PGD, PTSD, and depression total scores) later in time, separate weighted ANOVAs were conducted, using the BCH approach [36, 37]. We also explored what percentage of people met criteria for provisional diagnoses of PGD and PTSD at T2, and what percentage passed the threshold for clinically relevant depression at T2, and whether these percentages differed between participants included in the classes emerging at T1. People met criteria for probable PGD when the separation distress symptom, the functional impairment symptom, and five of nine additional symptoms from the PGD scale were endorsed with a > 3 response. People met criteria for probable PTSD as per DSM-IV when they scored 2 or 3 on at least one reexperiencing, three avoidance, and two hyperarousal symptoms [38]. The threshold for relevant depression was > 7 on the HADS depression scale [30].

Further correlates of class membership were calculated using the maximum-likelihood correction method [39]. We used distinct analyses to examine if classes differed in terms of (concurrently assessed) symptom levels of PGD, PTSD, and depression, socio-demographic (i.e. gender, age, and dichotomized educational level) and loss-related variables (i.e. kinship to the deceased [loss of partner/child vs. other losses], dichotomized cause of death, and time since loss), and the seven cognitive behavioural variables. Next, all variables that were significantly related with class membership in univariate analyses were included in one final multivariate model to examine which of these variables were most strongly correlated with class membership, when taking into account the shared variance between these variables. For each pairwise comparison, 95% confidence intervals (CI’s) were computed. When zero was not included in the 95% CI’s, the difference was considered significant.

## Results

### Latent class analysis

Table 1 shows fit indices for the one to six class solutions. Based on the fit indices and the interpretability of the class solutions, the three-class solution was selected as the optimal solution. This class had the lowest BIC, which is generally the preferred fit index to rely on [40]. The BIC is particularly preferred when BLRt *p* values are indecisive, as in our analyses, where all BLRt *p* values did not reach significance [41]. The four-class and five-class solutions yielder lower AICs and SS-BICs; however, the classes included small subsamples and the interpretability of these solutions was difficult. Hence, the three-class solution was retained.

**Table 1:** Goodness-of-fit statistics for one- through six-class solutions

	LL	AIC	BIC	SA-BIC	Entropy *R*^2^	*p* value BLRt	Smallest sample size
One class	− 4277.51	8601.02	8687.83	8614.88			–
Two classes	− 3380.53	6855.05	7032.45	6883.38	0.94	0.08	142
Three classes	− 3187.64	6517.29	6785.28	6560.08	0.92	0.42	96
Four classes	− 3122.85	6435.7	6794.28	6492.95	0.90	0.51	45
Five classes	− 3075.77	6389.53	6838.71	6461.25	0.89	0.51	31
Six classes	− 3045.16	6376.32	6916.08	6462.51	0.90	0.55	10

*AIC* Akaike information criterion, *BIC* Bayesian information criterion, *BLRT* bootstrapped likelihood ratio test, *LL* log-likelihood, *LMR LRT* Lo–Mendell–Rubin likelihood ratio test, *SS-BIC* sample size-adjusted Bayesian information criterion

**Fig. 1:**
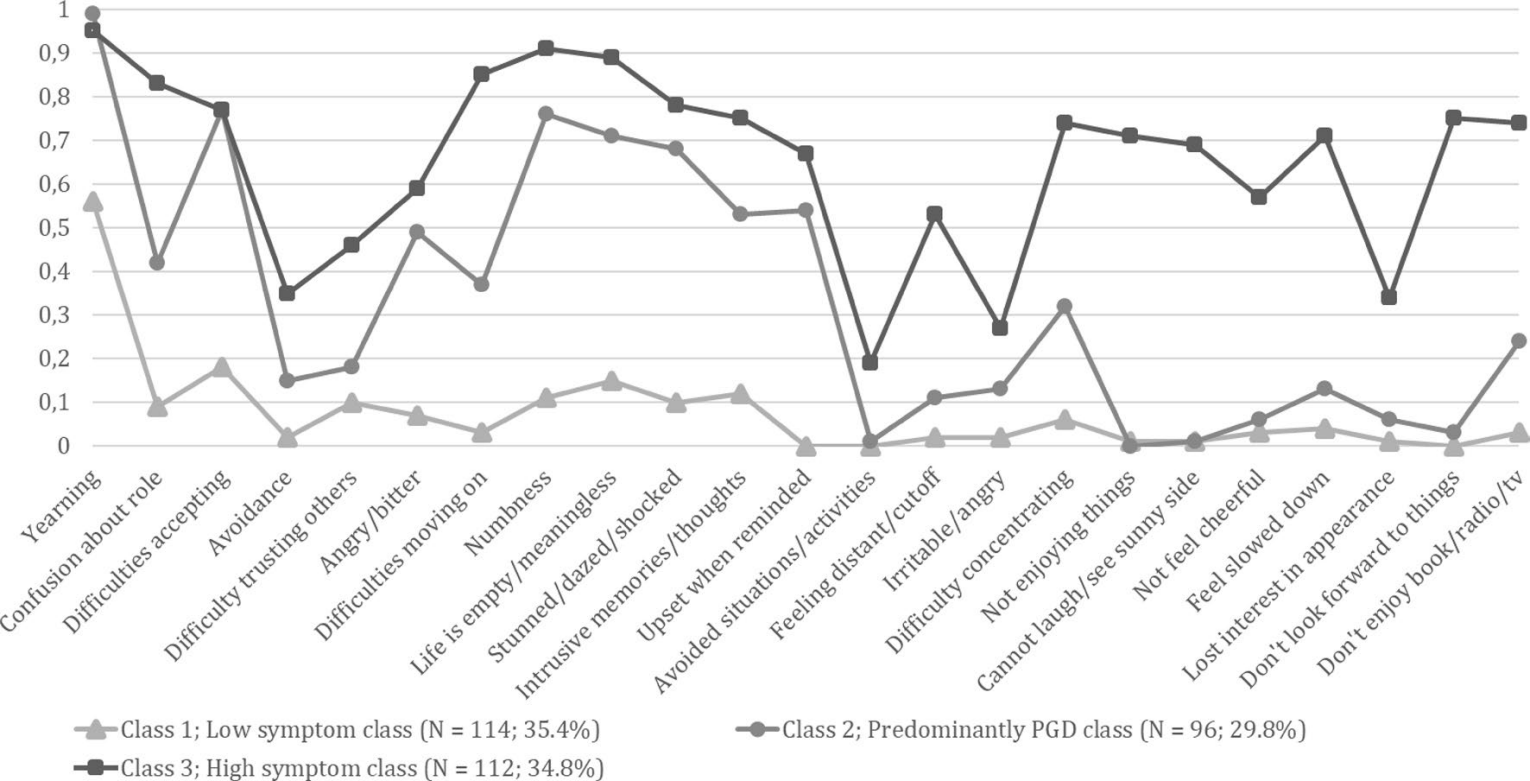
Plot probability estimates of a three-class solution

The symptom probabilities for the three-class solution are depicted in Fig. 1. Table 2 shows symptom frequencies in the total sample and probabilities of symptom endorsement for each class. We considered values of ≥ 0.60 as representing high, values ≤ 0.59 and ≥ 0.16 as representing moderate, and values of ≤ 0.15 as representing low symptom probabilities [20]. Taken together, the solution comprised a low symptom class, a PGD class, and a high symptom class. The largest class included 114 people (35.4%) and was characterized by low probabilities of all symptoms, except difficulties accepting the loss (moderate probability) and yearning (moderate to high probability). This class was labeled as “low symptom class”. The second class included 96 people (29.8%) and was characterized by low probabilities of three of six PTSD symptoms, and six of seven depression symptoms, moderate probability of three PTSD, one depression, and four of ten PGD symptoms, and high probabilities of five of ten PGD symptoms. This class was labeled the “predominantly PGD class”. The third class included 112 people (34.8%) and was characterized by moderate probabilities of 3 of 10 PGD symptoms and 3 of 6 PTSD symptoms, and 2 of 7 depression symptoms, and high probabilities of all other 15 symptoms. This class was labeled the “high symptoms class”.

**Table 2:** Probability of item endorsement for symptoms for three-class solution

	Overall symptom frequency		Class 1: low symptom class (*n* = 114); 35.4%		Class 2: predominantly PGD class (*n* = 96); 29.8%		Class 3: high symptom class *(n* = 112); 34.8%	
	*N*	%	Probability	SE	Probability	SE	Probability	SE
Prolonged grief symptoms								
Yearning	266	82.9	0.56	0.05	0.99	0.01	0.95	0.02
Confusion about role	144	45.1	0.09	0.03	0.42	0.06	0.83	0.04
Difficulties accepting	181	56.6	0.18	0.04	0.77	0.05	0.77	0.04
Avoidance	56	17.6	0.02	0.02	0.15	0.04	0.35	0.05
Difficulty trusting others	80	25.0	0.10	0.03	0.18	0.04	0.46	0.05
Angry/bitter	121	37.9	0.07	0.03	0.49	0.06	0.59	0.05
Difficulties moving on	134	41.9	0.03	0.02	0.37	0.06	0.85	0.04
Numbness	188	58.8	0.11	0.03	0.76	0.05	0.91	0.03
Life is empty/meaningless	185	57.8	0.15	0.04	0.71	0.05	0.89	0.03
Stunned/dazed/shocked	164	51.6	0.10	0.03	0.68	0.05	0.78	0.04
Posttraumatic stress symptoms								
Intrusive memories/thoughts	147	46.2	0.12	0.03	0.53	0.05	0.75	0.04
Upset when reminded	128	40.0	0.00	0.00	0.54	0.06	0.67	0.05
Avoided situations/activities	23	7.2	0.00	0.00	0.01	0.01	0.19	0.04
Feeling distant/cutoff	71	22.6	0.02	0.01	0.11	0.04	0.53	0.05
Irritable/angry	45	14.2	0.02	0.01	0.13	0.04	0.27	0.04
Difficulty concentrating	121	38.3	0.06	0.02	0.32	0.06	0.74	0.04
Depression symptoms								
Not enjoying things	81	25.2	0.01	0.01	0.00	0.00	0.71	0.05
Cannot laugh/see sunny side	80	24.8	0.01	0.01	0.01	0.01	0.69	0.05
Not feel cheerful	73	22.7	0.03	0.02	0.06	0.03	0.57	0.05
Feel slowed down	97	30.2	0.04	0.02	0.13	0.04	0.71	0.04
Lost interest in appearance	45	14.1	0.01	0.01	0.06	0.02	0.34	0.05
Do not look forward to things	87	27.4	0.00	0.00	0.03	0.02	0.75	0.05
Do not enjoy book/radio/tv	111	34.6	0.03	0.02	0.24	0.05	0.74	0.04

#### Associations of class membership with concurrently assessed PGD, PTSD, and depression severity

Table 3 shows mean scores of overall PGD, PTSD, and depression (i.e. summed scores on the PGD scale, PSS-SR, and HADS depression scale, respectively) for the total sample and each class. Outcomes of three distinct weighted logistic regression analyses and pairwise comparisons, comparing total PGD, PTSD, and depression scores separately between classes are shown in Appendix A. These scores differed significantly between all three classes and were ordered such that low symptom class < predominantly PGD class < high symptom class.

**Table 3:** Univariate associations of class membership with PGD, PTSD, and depression severity

	Total sample	Class 1: low symptom class (*n* = 114); 35.4%	Class 2: predominantly PGD class (*n* = 96); 29.8%	Class 3: high symptom class (n = 112); 34.8%	Pairwise comparisons (class)
		M (SD)	M (SD)	M (SD)	
Concurrent symptoms levels					
PGD symptom severity	27.21 (9.80)	16.67 (3.78)	29.37 (5.33)	36.10 (6.39)	1 < 2 < 3
PTSD symptom severity	14.48 (9.53)	5.39 (4.31)	14.96 (5.66)	23.33 (7.23)	1 < 2 < 3
Depression symptoms severity	6.16 (5.20)	1.60 (1.80)	4.59 (2.63)	12.16 (3.02)	1 < 2 < 3
Symptom levels at Time 2					
PGD symptom severity (*n* = 159)	24.15 (9.48)	14.27 (3.43)	25.27 (6.99)	31.52 (7.66)	1 < 2 < 3
PTSD symptom severity (*n* = 157)	11.92 (8.17)	4.40 (3.60)	11.51 (5.91)	18.63 (7.11)	1 < 2 < 3
Depression symptoms severity (*n* = 157)	5.40 (4.77)	1.50 (1.89)	4.46 (3.60)	9.57 (4.19)	1 < 2 < 3
Socio-demographic and loss-related correlates					
Gender = woman, *N* (%)	237 (73.6)	73 (64.0)	72 (75.0)	92 (82.1)	1 < 3; 1 = 2; 2 = 3
Age (in years), M (SD)	55.46 (14.03)	58.86 (12.29)	56.96 (14.50)	50.71 (14.11)	1 = 2 > 3
Education = college/university, *N* (%)	176 (54.7)	67 (58.8)	46 (47.9)	63 (56.3)	1 = 2 = 3
Kinship = partner/child, *N* (%)	171 (53.1)	41 (36.0)	59 (61.5)	71 (63.4)	1 < 2 = 3
Cause of loss = unnatural, *N* (%)	28 (8.7)	4 (3.5)	9 (9.4)	15 (13.4)	1 = 2; 1 < 3; 2 = 3
Time since loss in months, M (SD)	3.40 (1.59)	3.69 (1.39)	3.11 (1.56)	3.35 (1.76)	1 > 2 = 3
Cognitive behavioural variables					
Unrealness (*n* = 322)	24.96 (11.73)	15.48 (10.61)	28.64 (9.10)	31.47 (8.10)	1 < 2 < 3
Negative cognitions about self (*n* = 321)	9.06 (5.49)	6.57 (2.80)	7.80 (3.31)	12.65 (6.99)	1 < 2 < 3
Negative cognitions about life (*n* = 321)	7.22 (5.11)	4.64 (2.66)	6.28 (3.98)	10.65 (5.92)	1 < 2 < 3
Negative cognitions about future (*n* = 320)	11.33 (5.88)	8.42 (4.11)	10.63 (5.23)	14.92 (6.12)	1 < 2 < 3
Catastrophic misinterpretations (*n* = 321)	9.58 (5.38)	5.87 (2.85)	9.30 (4.62)	13.64 (5.14)	1 < 2 < 3
Depressive avoidance (*n* = 322)	15.36 (10.17)	7.34 (4.88)	13.32 (7.13)	25.27 (7.90)	1 < 2 < 3
Anxious avoidance (*n* = 322)	11.08 (6.42)	7.54 (4.96)	11.33 (5.46)	14.46 (6.64)	1 < 2 < 3

*PGD* prolonged grief disorder, *PTSD* posttraumatic stress disorder

#### Associations of class membership with overall PGD, PTSD, and depression severity assessed at follow-up

Table 3 also shows mean total PGD, PTSD, and depression scores from the subgroup with available data at T2, about 6 months after T1 (*N* = 159, 158, and 157 for PGD, PTSD, and depression scores, respectively). Outcomes of three distinct weighted ANOVAs and pairwise comparisons are shown in Appendix A. Analyses showed that mean PGD, PTSD, and depression scores at T2 differed significantly between all three classes such that low symptom class < predominantly PGD class < high symptom class. Within the sample at T2 (*N* = 159), seven people (4.4%) met criteria for a provisional diagnosis of PGD, none of which were in the low symptom class, one of which was in the PGD class, and six of which were in the high symptom class. Of all 158 people at T2 with available PTSD data, 11 (7.0%) met criteria for a provisional PTSD diagnosis; none in the low symptom, 1 in the PGD, and 10 in the high symptom classes. Of all 157 people with depression data at T2, 53 (33.8%) passed the threshold for clinically relevant depression; none from the low symptom class, 11 from the PGD class, and 42 from the high symptom classes. Differences between classes in terms of percentages of PGD caseness, PTSD caseness, and clinical relevant depression at T2 were statistically significant (PGD: Chi square = 8.38, DF = 2, *N* = 159, *p* = 0.015; PTSD: Chi square = 15.83, DF = 2, *N* = 158, *p* < 0.001; depression: Chi square = 70.88, DF = 2, *N* = 157, *p* < 0.001).

#### Associations of class membership with socio-demographic and loss-related variables

Table 3 summarizes the outcomes of statistical tests, testing differences between classes in terms of each distinct socio-demographic and loss-related variable. The details are shown in Appendix A. There were significant differences in terms of gender and age, such that the high symptom class included more women and younger participants compared to the other classes (with no other differences between these classes). In addition, classes differed in terms of kinship (fewer people lost a partner or child in the low symptom class compared to the other classes), cause of loss (more people were confronted with unnatural losses in the high symptom class), and time since loss (more time had passed for people in the low symptom class).

#### Associations of class membership with cognitive behavioural variables

As shown in Table 3 (and Appendix A) distinct weighted logistic regression analyses showed that classes differed in terms of all cognitive behavioural variables. The endorsement of these variables was straightforwardly ordered as: low symptom level class < predominantly PGD class < high symptom class. This means that a sense of unrealness, all negative cognitions, and both forms of avoidance were lowest in the low symptom class, significantly higher in the predominantly PGD class, and highest in the high symptom class.

### Multivariate analysis

In a multivariate model, we examined which of the significant correlates (i.e. gender, age, kinship, time since loss, plus all cognitive behavioural variables) continued to predict class membership when accounting for the shared variance between these correlates. Results are summarized in Table 4. The predominantly PGD class and high symptom class differed from the low symptom class such that these more disturbed classes included more people confronted with the death of a partner or child, people that were more recently bereaved, and people reporting higher scores for unrealness, catastrophic misinterpretations, and depressive avoidance. Depressive avoidance was stronger in the high symptom class compared to the predominantly PGD class, and the only variable distinguishing these two classes.

**Table 4:** Multivariate model including socio-demographic, loss-related, and cognitive behavioural variables predicting class membership (*N* = 319)

	B	SE (B)	95% confidence interval	
Class 1 (low symptom class) vs. Class 2 (predominantly PGD class)				
Gender (0 = man, 1 = woman)	− 0.19	0.71	− 1.59	1.21
Age (in years)	0.01	0.02	− 0.03	0.06
Kinship (0 = other. 1 = child/spouse)	**1.81**	0.70	0.43	3.19
Time since loss (in months)	− **1.07**	0.38	− 1.80	− 0.33
Unrealness	**0.15**	0.03	0.08	0.21
Negative cognitions about self	− 0.06	0.11	− 0.29	0.16
Negative cognitions about life	0.19	0.17	− 0.15	0.52
Negative cognitions about future	− 0.14	0.11	− 0.36	0.07
Catastrophic misinterpretations	**0.20**	0.08	0.05	0.35
Depressive avoidance	**0.33**	0.09	0.14	0.51
Anxious avoidance	− 0.05	0.07	− 0.18	0.08
Class 1 (low symptom class) vs. Class 3 (high symptom class)				
Gender (0 = man. 1 = woman)	0.54	0.99	− 1.40	2.47
Age (in years)	− 0.02	0.03	− 0.07	0.03
Kinship (0 = other. 1 = child/spouse)	**2.52**	0.85	0.85	4.19
Time since loss (in months)	− **1.05**	0.38	− 1.80	− 0.30
Unrealness	**0.16**	0.04	0.08	0.24
Negative cognitions about self	0.07	0.12	− 0.16	0.30
Negative cognitions about life	0.16	0.18	− 0.19	0.51
Negative cognitions about future	− 0.11	0.11	− 0.32	0.11
Catastrophic misinterpretations	**0.30**	0.08	0.14	0.46
Depressive avoidance	**0.50**	0.10	0.31	0.69
Anxious avoidance	− 0.09	0.07	− 0.23	0.06
Class 2 (predominantly PGD class) vs. Class 3 (high symptom class)				
Gender (0 = man. 1 = woman)	0.73	0.77	− 0.77	2.23
Age (in years)	− 0.03	0.02	− 0.07	0.01
Kinship (0 = other. 1 = child/spouse)	0.71	0.61	− 0.49	1.91
Time since loss (in months)	0.01	0.14	− 0.25	0.28
Unrealness	0.01	0.03	− 0.05	0.08
Negative cognitions about self	0.13	0.08	− 0.02	0.29
Negative cognitions about life	− 0.03	0.08	− 0.19	0.13
Negative cognitions about future	0.04	0.05	− 0.07	0.14
Catastrophic misinterpretations	0.10	0.05	0.00	0.21
Depressive avoidance	**0.17**	0.04	0.10	0.25
Anxious avoidance	− 0.04	0.04	− 0.12	0.05

In bold represent significant (*p* < 0.050) difference

## Discussion

The severity of initial reactions following the death of a loved one predicts the severity of later responses [6, 8]. Knowledge about patterns and correlates of early responses is scarce but important, because it may help to inform methods for the early identification and treatment of subgroups at risk for persistent distress. In a prior study, we examined patterns of early symptoms of PCBD symptoms (as per DSM-5 [4]) and found three classes, characterized by low PCBD symptoms, elevated separation distress symptoms, and severe PCBD symptoms, respectively [8]. The current study built on that prior work by examining whether subgroups could be identified among very recently (≤ 6 months) bereaved individuals, based on their endorsement of symptoms of PGD (as per Prigerson et al. [3]) as well as PTSD and depression. Furthermore, associations of class membership with overall disturbed grief, PTSD, and depression assessed concurrently and at 6 months follow-up were examined. Finally, we explored differences between classes in terms of socio-demographics, loss-related, and cognitive behavioural variables.

A first main finding was that three subgroups could be discerned, one group with low symptom levels across all symptom domains (except “yearning”, endorsed by 56% of people), a second group with elevated probabilities of endorsing most PGD symptoms (but few other symptoms), and a third group with high scores on almost all symptoms. Prior research examining latent classes of more remotely bereaved individuals (i.e., > 6 months post-loss) has also identified classes differing in the nature of symptoms, including classes with elevated PGD (but not other symptoms) and classes characterized by elevations in combinations of symptoms (e.g. [19]). Our findings accords with these prior findings and suggest that, even in the first half year of bereavement, a subgroup exists with people suffering from elevated PGD symptoms, but not other symptoms. That “yearning” was strongly endorsed in the low symptoms class accords with the notion that yearning is one of the most commonly reported affective experience following loss [42, 43]. That the second class stood out in terms of the endorsement of symptoms of PGD as put forth by Prigerson et al. [3] and not PTSD and depression symptoms supports the notion that these particular symptoms represent a coherent syndrome that is distinguishable from other forms of bereavement-related distress.

A second main finding was that symptom levels of overall disturbed grief, PTSD, and depression (assessed concurrently and approximately 6 months later) were lowest in the low symptom class, higher in the predominantly PGD class, and highest in the high symptom class. Outcomes of the analyses with class membership predicting scores above meaningful cutoffs mirrored these findings: people in the predominantly PGD class and (even more so) people in the high symptom classes had higher chances of meeting criteria for probably PGD, PTSD, and elevated depression at follow-up. This indicates that differentiating recently bereaved people based on their early responses has concurrent and predictive validity. That people with elevated PGD only were already at risk for elevated symptom levels 6 months later suggests that detecting and treating these people may have preventative effects.

With respect to socio-demographic and loss-related correlates, a third main finding was that people who were woman, younger, more recently bereaved, bereaved by the loss of a partner or child, and bereaved due to an unnatural loss had a larger chance of being in the high symptom class compared to the other classes. In our final analyses, we examined differences between classes in terms of cognitive behavioural variables [10]. A fourth main finding was that the endorsement of these variables—a sense of “unrealness” about the loss, negative cognitions about the self, life, the future, and one’s grief, plus anxious and depressive avoidance tendencies—was lowest in the low symptom level class, intermediate in the predominantly PGD class, and highest in the high symptom class. These findings are consistent with cognitive behavioural theories of disturbed grief [10, 22] and with prior evidence that subgroups of bereaved people with more pervasive grief reactions tend to engage in maladaptive cognitive and behavioural patterns [8, 15, 20].

Multivariate analyses showed that the predominantly PGD and high symptom classes differed from the low symptom class such that these two classes included more people confronted with the death of a partner or child, were more recently bereaved, and more strongly inclined to experience the loss as “unreal”, to endorse catastrophic misinterpretations about their own grief, and to engage in depressive avoidance. Depressive avoidance was the only variable distinguishing the predominantly PGD from the high symptom class; this suggests that withdrawing from usual activities, even in the very early months of bereavement, contributes to different forms of distress following loss [8, 22]. That depressive avoidance was important in distinguishing classes, whilst avoidance as a symptom of PGD was not strongly endorsed may be due to the fact that depressive avoidance refers to passivity driven by a lack of motivation, whereas the PGD symptom of avoidance refers to an anxiety-driven avoidance of loss-related cues.

Findings should be considered in light of several limitations. Firstly, in this study we focused on PGD symptoms according to Prigerson et al. [3]. There is evidence that these criteria overlap with PGD criteria proposed for ICD-11 [44] and criteria for PCBD [4]. However, some caution should be applied in generalizing our finding to people with early disturbed grief based on other criteria sets. Secondly, the majority of our sample was women, middle aged, and bereaved after a natural loss. It remains to be studied if our findings may be replicated in other bereaved samples. Thirdly, self-report measures were used to assess symptoms of PGD, PTSD, and depression which may lead to overestimations of symptom severity levels. Fourthly, our analyses concerning differences in cognitive behavioural variables between classes were based on cross-sectional data and, therefore, do not allow conclusions about the reciprocal associations between variables. Thus, whether early depressive avoidance precedes elevated comorbid symptoms, or vice versa, in the first half year of bereavement, still remains to be studied, using longitudinal designs.

Notwithstanding these limitations, the current study expands our knowledge by showing that, in the first 6 months of bereavement, meaningful subgroups of bereaved people can be distinguished—with low symptoms, elevated PGD symptoms, and elevated distress across different symptom domains. Relevant to a clinical staging perspective, our findings suggest that elevated symptoms of PGD, traumatic stress, and depression in this period not always reflect transient distress, but could be harbingers of chronic bereavement-related distress [45]. This is important because identifying people with increased PGD and other symptoms in this particular time frame and offering them interventions to take away obstacles blocking the resolution of symptoms may have significant preventative effects.

## Appendix A

See Table 5

**Table 5:** Correlates of class membership in univariate testing

	Class 1 (low symptoms) vs. Class 2 (predominantly PGD)				Class 1 (low symptoms) vs. Class 3 (high symptoms)				Class 2 (predominantly PGD) vs Class 3 (high symptoms)				
	B	SE	95% CI		B	SE	95% CI		B	SE	95% CI		Overall *p* value
Concurrent symptoms levels													
PGD symptom severity	0.73	0.10	0.54	0.92	1.00	0.12	0.77	1.23	0.27	0.05	0.16	0.38	< 0.001
PTSD symptom severity	0.63	0.11	0.42	0.83	0.87	0.11	0.65	1.10	0.25	0.04	0.17	0.32	< 0.001
Depression symptoms severity	0.77	0.10	0.58	0.96	2.51	0.52	1.49	3.52	1.74	0.50	0.76	2.72	< 0.001
Prospective symptom levels													
PGD symptom severity (*N* = 159)	11.51	1.10	9.36	13.66	17.75	1.14	15.52	19.99	6.25	1.44	3.42	9.07	< 0.001
PTSD symptom severity (*N* = 157)	7.44	1.00	5.49	9.39	14.79	1.09	12.66	16.92	7.35	1.30	4.79	9.90	< 0.001
Depression symptoms severity (*N* = 157)	2.94	0.60	1.77	4.11	8.26	0.63	7.04	9.49	5.32	0.77	3.82	6.83	< 0.001
Socio-demographic and loss-related correlates													
Gender (0 = men, 1 = women)	0.55	0.33	− 0.09	1.19	0.98	0.32	0.35	1.61	0.43	0.36	− 0.27	1.13	0.008
Age (in years)	− 0.01	0.01	− 0.03	0.01	− 0.04	0.01	− 0.06	− 0.02	− 0.04	0.01	− 0.06	− 0.01	<0.001
Education (0 = other, 1 = university/college)	− 0.54	0.30	− 1.12	0.05	− 0.14	0.28	− 0.68	0.41	0.40	0.29	− 0.18	0.97	0.180
Kinship (0 = other, 1 = child/spouse)	1.34	0.31	0.73	1.96	1.21	0.29	0.65	1.77	− 0.13	0.30	− 0.73	0.46	< 0.001
Cause of loss (0 = natural, 1 = unnatural)	0.95	0.66	− 0.34	2.23	1.38	0.59	0.22	2.55	0.44	0.47	− 0.48	1.35	0.063
Time since loss (in months)	− 0.26	0.09	− 0.43	− 0.09	− 0.15	0.09	− 0.32	0.03	0.11	0.10	− 0.08	0.30	0.008
Cognitive behavioural variables													
Unrealness (*N* = 322)	0.13	0.02	0.09	0.17	0.17	0.02	0.13	0.21	0.04	0.02	0.01	0.07	< 0.001
Negative cognitions about self (*N* = 321)	2.08	0.81	0.49	3.67	2.28	0.81	0.69	3.87	0.20	0.06	0.07	0.32	< 0.001
Negative cognitions about life (*N* = 321)	2.65	1.19	0.32	4.97	2.81	1.18	0.49	5.13	0.17	0.04	0.09	0.24	< 0.001
Negative cognitions about future (*N* = 320)	0.13	0.04	0.05	0.22	0.28	0.04	0.19	0.36	0.14	0.03	0.08	0.20	< 0.001
Catastrophic misinterpretations (*N* = 321)	0.34	0.09	0.16	0.52	0.54	0.09	0.36	0.72	0.20	0.04	0.13	0.27	< 0.001
Depressive avoidance (*N* = 322)	0.30	0.06	0.19	0.41	0.50	0.06	0.37	0.62	0.20	0.03	0.14	0.26	< 0.001
Anxious avoidance (*N* = 322)	0.20	0.06	0.09	0.31	0.28	0.05	0.17	0.38	0.08	0.02	0.03	0.12	< 0.001

*PGD * prolonged grief disorder, *PTSD* posttraumatic stress disorder
